# An Algorithm for Jaw Pain among Divers

**DOI:** 10.3390/jcm13113167

**Published:** 2024-05-28

**Authors:** Angelo Vivacqua, Kathleen Fan, Alexander Gürtler, Florian M. Thieringer, Britt-Isabelle Berg

**Affiliations:** 1 Department of Oral and Cranio-Maxillofacial Surgery, University Hospital Basel, 4031 Basel, Switzerland; vivacqua@zahnarzt-stjohann.ch (A.V.); florian.thieringer@usb.ch (F.M.T.); 2 Oral and Maxillofacial Surgery Department, King’s College Hospital NHS Foundation Trust, London SE5 9RS, UK; kfan@nhs.net; 3 Physiothek, Private Practice, 4054 Basel, Switzerland; guertler@physiothek-basel.ch; 4 Faculty of Medicine, University Hospital Basel, 4031 Basel, Switzerland; 5 Medical Additive Manufacturing Research Group (Swiss MAM), Department of Biomedical Engineering, University Basel, 4001 Basel, Switzerland

**Keywords:** temporomandibular joint, masticatory muscles, diving, pain, osteoarthritis, decompression sickness

## Abstract

**Background:** Temporomandibular disease (TMD) is commonly seen, and divers also experience pain in the temporomandibular joint (TMJ) or masticatory muscles. This article aims to provide a tool for diving physicians or medical professionals involved in diving medicine since jaw pain among divers is a pertinent subject and can be challenging to evaluate without some background in dentistry or maxillofacial surgery. **Method:** A basic algorithm was developed to provide a tool to differentiate jaw pains experienced by divers. Three brief case studies were developed, and five diving physicians were tasked with diagnosing the cases using the algorithm. Additionally, simple exercises and massage techniques that can benefit patients with TMD, particularly immediately after diving, are outlined. **Results:** All five diving physicians successfully diagnosed the cases using the algorithm. However, three of them were unable to diagnose the first case (disc luxation) without consulting the algorithm. Nevertheless, all physicians acknowledged the utility of the algorithm. **Conclusions:** Jaw pain in divers can stem from diverse causes, but effective treatment options exist. Our study findings provide valuable insights to assist diving physicians in making accurate diagnoses and guiding appropriate patient management, which may include referrals to specialists such as dentists, maxillofacial surgeons, or orthodontists.

## 1. Introduction

The temporomandibular disorder (TMD) is very common in the general population, and divers are no exception in terms of experiencing pain in the temporomandibular joint (TMJ) or masticatory muscles.

Since TMD encompasses a broad spectrum of conditions, it is crucial to distinguish between various potential causes of pain. Decompression sickness affecting a small joint like the TMJ is an improbable source of pain. While reports of such occurrences exist in the literature concerning major joints such as the shoulder or knee [1,2], they are extremely rare in current publications [3].

Pain originating from a muscle or joint due to an ill-fitting mouthpiece is more prevalent [4,5]. Numerous publications emphasize the importance of using a properly fitted mouthpiece to maintain correct positioning in the mouth [4,5,6]. Damage to the mouthpiece makes it challenging to maintain the correct position, with a distal jaw position being unlikely due to human anatomy but a more anterior position being common. Misalignment of the jaw from its physiological position can result in TMJ pain, particularly after multiple dives where prolonged incorrect positioning occurs.

As the diving population ages, conditions commonly seen in the elderly, such as arthritis/osteoarthritis [7], become increasingly prevalent among divers. While arthritis is well-known for causing shoulder or knee pain, it can also affect joints like the TMJ. Divers who occasionally dive may not be aware of osteoarthritis until they spend periods biting on their mouthpiece underwater.

A dislocated disc can become more painful after diving compared to normal daily activities, where it experiences force primarily during chewing or clenching. Patients typically present themselves in outpatient clinics with pain, a clicking noise, or limited mouth opening [8], indicative of disc displacement. During the dive, there is one steady position. The mouth remains more open than in its physiological resting position. Although the distance is minimal, it can exert some impact on the joint. Pre-existing joint damage can lead to further dislocation and increased pain.

Overall, Aldridge and Fenlon reported a 47.6% incidence of at least one TMD symptom among scuba divers [9]. They suggested that the presence of multiple symptoms is necessary for diagnosing TMD syndrome [9]. However, even a single symptom can significantly affect a diver’s quality of life during or after a dive.

An increased risk of developing TMDs or a trigger for it can be assumed to be due to stressful times [10,11], which are due to a variety of reasons, including the coronavirus disease 2019 (COVID-19) lockdown and less experience in diving [12]. Depression, isolation, and other mental conditions, including anxiety or stress, can lead to the development of TMD symptoms through the “hypothalamic–pituitary–adrenal axis” [13]. De Paiva Tosato et al. [14] described that cortisol release leads to greater muscle activity and is positively correlated with the degree of TMD severity [14]. In women, TMD symptoms are more often seen not only after diving or while diving but also before diving [15], which was also reported by Aldridge and Fenlon, who studied a young group of divers [9]. In divers with previous TMD, biting for a longer time on the mouthpiece can increase the TMD symptoms. One of its causes could be the mouthpiece itself, and many studies have investigated the matter, focusing on individual mouthpieces [4,5,12]. 

This article aims to provide a tool for diving physicians or medical professionals involved in diving medicine. Algorithms are already employed in various settings and disciplines [16]. Jaw pain among divers is a pertinent subject and can be challenging to evaluate without some background in dentistry or maxillofacial surgery. This algorithm is intended to bridge this knowledge gap.

## 2. Materials and Method

**Figure 1:**
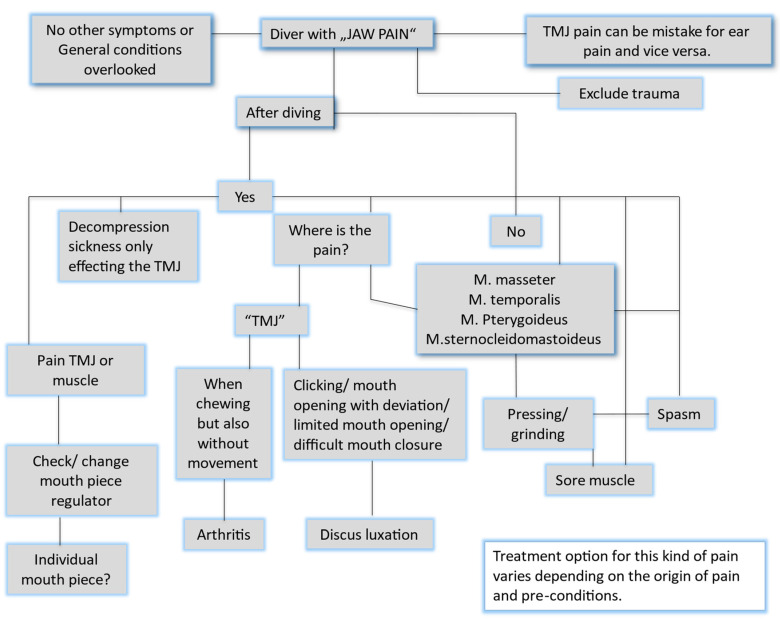
An algorithm to differentiate the causes of jaw pain among divers.

To assess the effectiveness of the algorithm, five diving physicians (three females and two males), none of whom had experience in maxillofacial surgery or dentistry, were asked to respond to the following three cases using the algorithm depicted below (Figure 1):

Case 1: A 42-year-old female diver reported experiencing a distinct clicking sensation in her jaw joint during her recent diving safari. She had previously noticed this issue a few years ago following an extensive dental treatment. What is the most likely diagnosis? Correct answer: disc displacement.

Case 2: A 19-year-old, inexperienced diver complains of experiencing pain in his chewing muscles during his 5-day diving course, particularly in the evening. His girlfriend did not observe any grinding during the night but noted slight snoring instead. What is the most likely diagnosis? Correct answer: masticatory muscle pain.

Case A 65-year-old female diver describes experiencing an itchy sensation in the upper part of her body and a sharp pain in her left TMJ after her last dive. There are no audible clicking sounds, and there is no history of TMJ pain. What diagnosis should be considered? Correct answer: decompression sickness.

The physicians were also asked if they could answer the questions without the algorithm and whether they found the algorithm helpful. 

The answers were collected in an Excel spreadsheet.

## 3. Results

All five diving physicians accurately diagnosed the cases using the algorithm. Three of them admitted that they would not have been able to answer the first case (disc luxation) without relying on the algorithm. Moreover, all physicians unanimously agreed on the utility of the algorithm.

## 4. Discussion

We have demonstrated the effectiveness of the algorithm in aiding physicians who lack experience with jaw pain in divers. Our findings reveal that more than 50% of the participants would not have been able to identify one of the causes of this type of jaw pain (disc luxation), which is a prevalent jaw pathology [7], without the assistance of the algorithm.

Once the diver is consulting a physician, the next step is to identify when the pain occurs. Pain can occur in daily life and exaggerate after diving, or it may appear for the first time after diving. In these cases, the following causes need to be considered.

### 4.1. Decompression Sickness (DCS)

DCS in the TMJ is extremely rare. TMJ pain can be a symptom of DCS. Differentiating it from other causes is difficult [3]. DCS mostly occurs in larger joints such as the knees, shoulders, or elbows [17]. The symptoms include pain, which can be due to nitrogen bubbles in the tissues owing to less gas elimination after diving. At the time of completing the present paper, only one publication was available that reported the possible causes of having only TMJ pain as a symptom of DCS [3]. One reason could be that there is no way to differentiate the DCS of a TMJ from the first episode of a non-DCS TMJ pain event, except to send the patient into a hyperbaric chamber. As this phenomenon is uncommon, divers will consider that their pain is due to another cause, except for DCS. If more DCS symptoms occur, a hyperbaric chamber treatment will be effective for the TMJ pain. In theory, it might be possible to detect bubbles that cause pain in the TMJ using ultrasound (US). Publications on bubbles in vessels support this notion [18]. However, in practise, the US of the TMJ is primarily employed for visualizing disc movement and position [19]. Due to the small size of the bubble, US may not have the capability to capture them effectively.

The other pains of the jaw after diving can have a muscular or TMJ origin or both. Thus, it is necessary to determine whether there have been previous episodes. If this is not the case, mouthpiece assessment is important.

### 4.2. Mouthpiece-Related Pain

**Figure 2:**
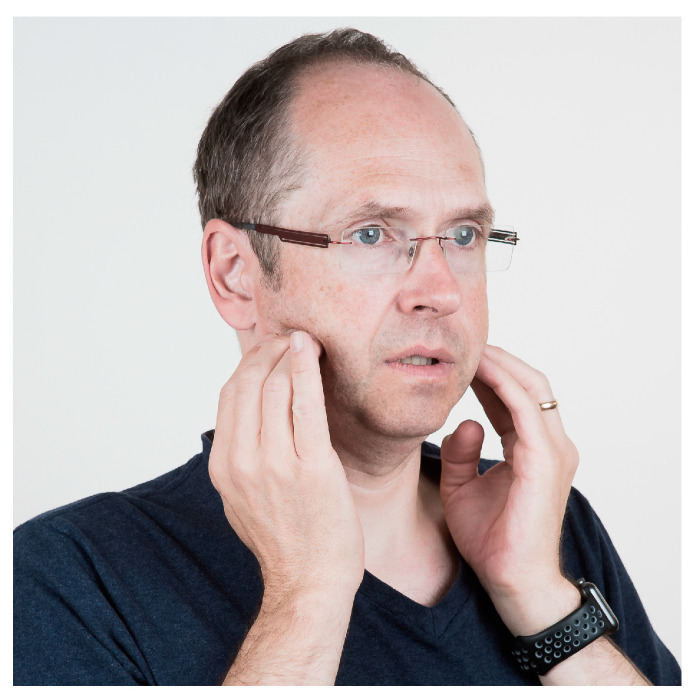
Relaxation of the m. masseter. Massage of the m. masseter in the cheek area. Massage the attachment points in particular. Stroking the muscle strands or circling the pain point is recommended. The pressure of the techniques is best chosen so that the pain threshold is not exceeded, and the targeted pain points can be massaged. At the same time, the lower jaw should be slightly open without tension.

**Figure 3:**
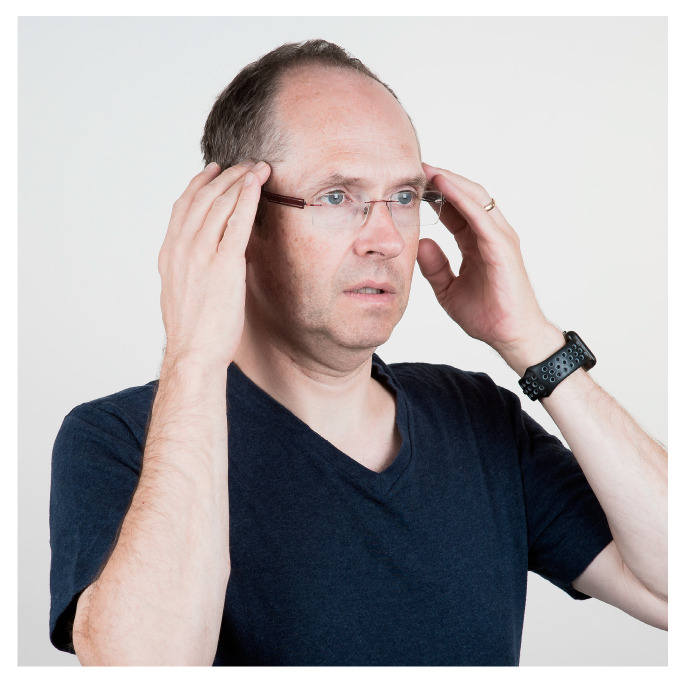
Relaxation or massage of the m. temporalis. Massage the temporal region with your fingers. We recommend stroking the muscle strands or circling the pain point. The pressure of the techniques is best chosen so that the pain threshold is not exceeded, and the targeted pain points can be massaged three times a day for 2–3 min each. At the same time, the lower jaw should be slightly open without tension.

**Figure 4:**
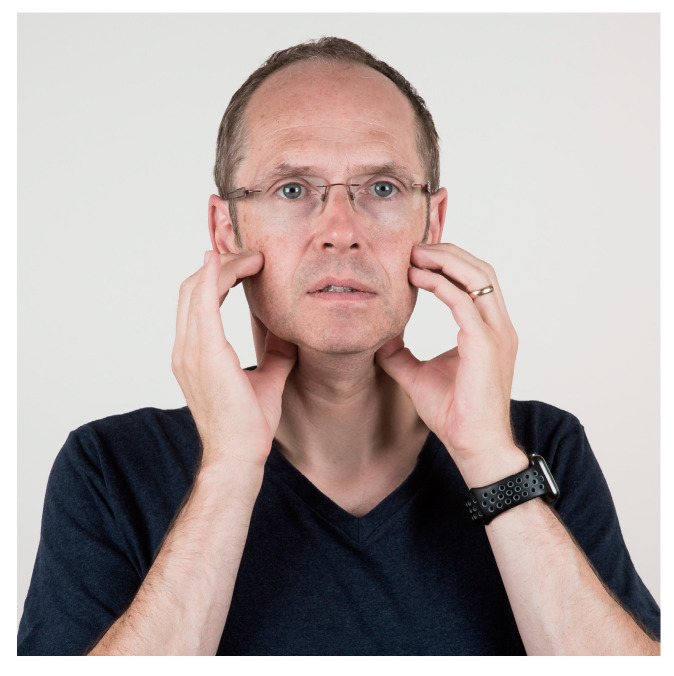
Relaxation or massage of the medial pterygoideus muscle. The inside of the lower jaw should be pressed outwards and massaged lightly. We recommend stroking the muscle strands or circling the pain point. The pressure of the techniques is best chosen so that the pain threshold is not exceeded, and the targeted pain points can be massaged. At the same time, the lower jaw should be slightly open without tension.

**Figure 5:**
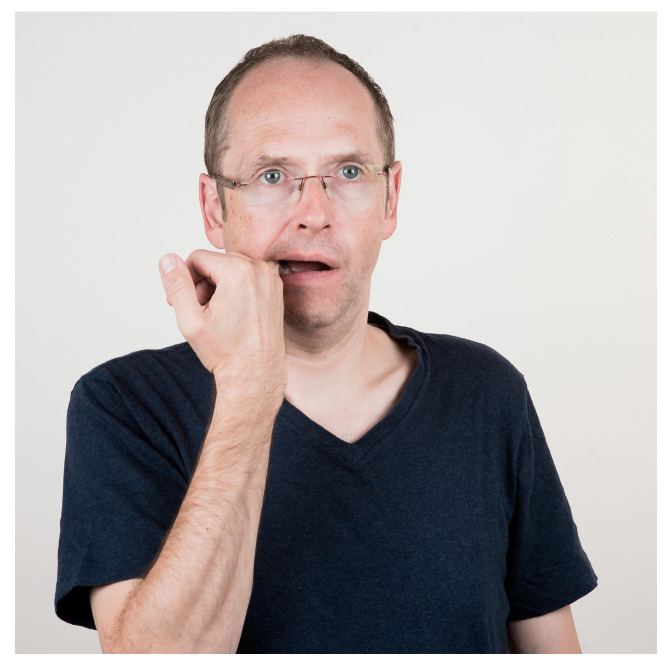
Relaxation or massage of the lateral pterygoid muscle. The area along the upper row of teeth should be pressed gently backward or upward with your finger. Press carefully, as this muscle is often painful. At the same time, the lower jaw should be slightly open without tension.

Many studies have found associations between TMJ and muscle pains and mouthpieces [4,5]. To maintain the correct positive position of the mouthpiece, it sometimes requires some muscular effort. Muscular fatigue can be a consequence of the forces used to keep the mouthpiece in place, especially in strong currents when the lip muscle strength and light bite forces might not be sufficient. Mouthpiece use also requires muscle strength to balance the regulator due to its weight. Although underwater the weight seems to be less, it is an uncommon position for divers since it is not a physiological position. Especially for divers who do not dive regularly, this can lead to problems. Thus, care should be taken not to bite too hard on the mouthpiece. Otherwise, the mastication muscles will be overloaded, which can lead to muscle pain due to muscle tension. Diving is no different from other sports. Specific muscles are being built up. It is not uncommon that divers will spend a week on a safari boat, diving 3–4 times a day for up to 60 min per dive, leading to overstraining of the muscles, which are not frequently used in this manner in daily life. The pain felt in the muscles, especially m. masseter, temporalis, and pterygoid medialis/lateralis, can be managed with gentle massage that reduces muscle soreness (Figure 2, Figure 3, Figure 4 and Figure 5). All the exercises shown should be repeated 2–3 min after brushing the teeth in the morning and evening.

For severe pain immediately after diving, the application of cold packs and, if not available, a plastic bag filled with ice cubes and covered with a thin towel can decrease swelling. Owing to the cold compress, the blood vessels constrict. Extremely cold packs also cause the contracted vessels to dilate even more when a normal temperature is achieved, which can increase the swelling. However, it is important to have breaks in between the cooling applications and not to put the ice straight on the skin because this can lead to frostbite. Some patients do prefer hot packs on the strained muscles or, if not available, a small towel that is wrung out in hot water. The heat will increase circulation and can also help to reduce the pain, but if an inflammatory process is present, it can worsen the situation [20].

Most mouthpieces are made from silicone or neoprene. The common characteristic among mouthpieces is that they all have an interdental bite platform. When divers are strongly clinching, the interdental part of the mouthpiece will be damaged or torn, which can have an impact on the mouthpiece’s position and dive safety. A torn in the interdental bite platform will lead to an imbalance in the occlusal plate, thereby leading to an imbalance of the TMJs. Torn interdental platforms occur not only in diving beginners who might be stressed in shallow water and bite more forcefully than necessary but also in very experienced divers who might have bitten the mouthpiece forcefully to avoid a loss of the regulator in the current. Therefore, it should always be checked regularly if a replacement of this part of the regulator is necessary. TMJ pain due to an unusual mandible position can occur, too. To keep the mouthpiece in the correct position, the mandible is often not in the normal physiological position but is positioned further forward. This leads to a lack of support in the molar region, which can end in TMJ pain [21].

Patients with TMJ pain can experience discomfort, pain, and a locked joint; thus, the cause of the pain should be determined. If the mouthpiece’s shape or size causes the TMDs, using an individualized mouthpiece could reduce the symptoms. Exercises with professional help (physiotherapy) are similar to those used by chronic pain patients. Moreover, exercises performed by the divers at home can have a remarkable impact.

The influence of water temperature on TMD is controversial. Lobbezoo et al. suggested that diving in cold water might prevent TMD pain [22], but Aldridge and Fenlon have shown that more divers reported TMD symptoms in cold water than in warm water. Jaw stiffness is a suspected reason for this phenomenon [9]. Nevertheless, divers with TMD or mouthpiece-triggered TMD can still develop symptoms in warm or cold water.

In most cases, diving physicians are not always with the divers directly after the dive. More often, the diver will seek advice from a diving physician or dentist about the pain once he is back home. In this setting, achieving an accurate diagnosis would depend on the diving physician’s experience.

The algorithm (Figure 1) should function as a simple tool to roughly differentiate the different causes of jaw pain, including muscle and TMJ pains, which will persist. Diving can exaggerate the different types of pain, which can become a chronic issue.

The primary question requiring an answer is where the pain occurs. If it is in the TMJ, ear pain and a traumatic reason must be excluded. The TMJ is directly adjacent to the ear. The discomallear ligament is described as a connection between the malleus (of the ear) and medial joint capsule. However, it seems that an isolated ligament can only be detected in 29% of TMJs [23]. Ear pain, if there is no sign of an ear infection, can also result from myofacial trigger points or muscular tensions of the m. masseter [24]. If ear infections occur, tension can also occur in the TMJ. Traumatic reasons can include fractures, but a strong contusion can also cause edema, followed by pain. Traumatic events in the past also need to be excluded. Especially if an accident happened in cold water, divers might not be consciously aware of it. Moreover, trauma in adolescence and even in childhood can have an impact on the development of later TMDs.

### 4.3. Arthritis/Osteoarthritis

Constant pain after diving, but also in daily life without problems with mouth opening, especially if a pre-auricular swelling is visible, might be caused by arthritis. These patients require radiological imaging. Further treatment depends on the severity of the condition. In some patients, administration of nonsteroidal anti-inflammatory drugs (NSAIDs) for a week (along with drugs to protect the stomach) and a strict soft diet can already bring some relief. Barjandi [25] et al.’s literature review demonstrated that naproxen and ibuprofen are the first choice of NSAIDs in patients at risk for cardiovascular diseases, and if the patient is at risk for gastrointestinal bleeding, celecoxib should be chosen [25].

Cold packs can also help stabilize the situation. Rinsing the joint is not the first choice of treatment, but it can also be an option. If more than one joint is affected, a rheumatologist should be consulted. Once the acute phase is managed, physiotherapy can also be useful.

The diagnosis can be visualized using various imaging modalities. Computed tomography (CT) [26], cone beam computed tomography (CBCT) [27], and panoramic imaging [28] are commonly utilized for this purpose, although they all involve exposure to radiation. Magnetic resonance imaging (MRI) is also a viable option, particularly in children, due to its radiation-free nature, but it has the drawback of longer acquisition times and a higher cost. 

Once the anamneses revealed no other general conditions, such as arthritis or osteoarthritis, the following differential diagnosis might be the reason for pain in the jaw.

### 4.4. TMJ Disc Displacement with or without a Reduction

Another cause of pain is TMJ disc displacement, with or without a reduction. It is characterized by a locked jaw, a limited mouth opening for disc replacement without a reduction and a clicking noise, and a deviation of the mouth opening for disc replacement with a reduction. 

To confirm the clinical diagnosis, US is a viable option [29], but MRI, particularly dynamic MRI [30], is frequently the preferred imaging modality. Additionally, for patients who experience claustrophobia, open MRI scanners are available, offering a more comfortable imaging experience.

Concerning the treatment, NSAIDs, along with stomach protection, a very soft-to-liquid diet, and physiotherapy, might bring some relief. Locally applied NSAID is also effective, but it will be washed off during diving; therefore, it is only advisable to apply this drug in the evening after the last dive.

**Figure 6:**
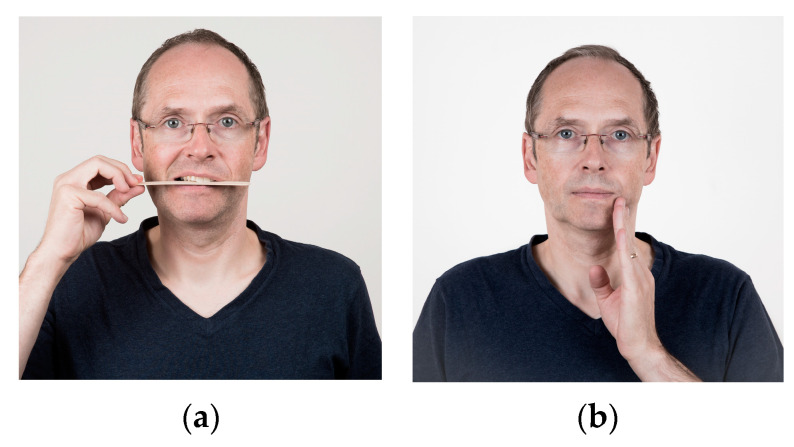
(**a**,**b**) Exercises to improve the lateral movement of the lower jaw. (**a**) Place your right hand on your right jaw. Gently push your jaw against the hand, increasing pressure of the hand. (**b**) Slowly move the lower jaw to your right and left. If necessary, a wooden stick, pencil, or a cork cone can also be held loosely between the teeth. Repeat this exercise five times.

**Figure 7:**
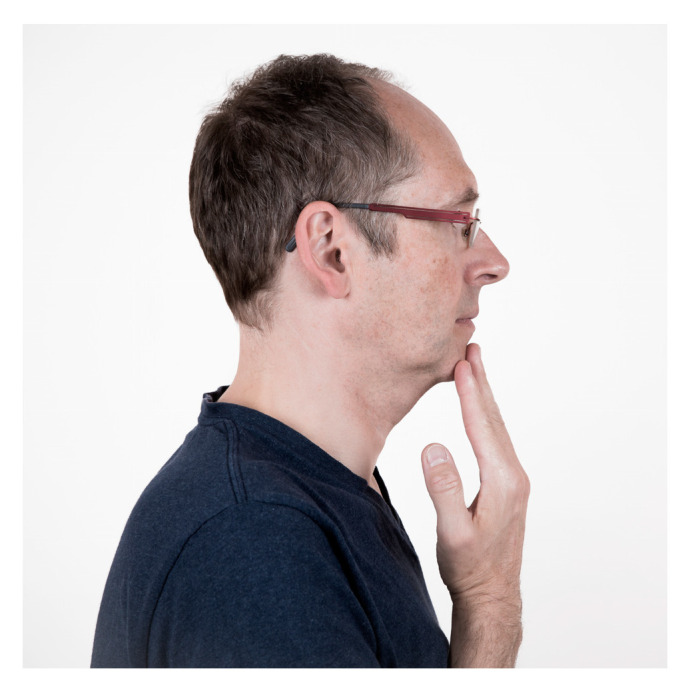
Forward and backward movements of the jaw. Touch the front of your chin with your fingers. Slowly tense the lower jaw forward and downward without any movement. Hold the tension for 10 s.

In some patients, the use of a Michigan splint is also useful. Others might even need an arthrocentesis. All of these procedures should be accompanied by physiotherapy. Figure 6 and Figure 7 are simple exercises that can be carried at home and on a dive boat. These exercises help improve jaw movement and reduce pain.

Studies on the usage of botulin toxin injections are also available [31]. Open surgery for disc repair or removal can be considered if all other options are not effective. Conservative treatment should always be the first choice.

### 4.5. Muscular Pain

Muscular pain can occur due to various reasons. Pressing, clinching, or grinding of the teeth leads to muscle soreness and spasm. In free divers, muscle tightness is commonly seen. The m. sternocleidomastoideus can affect TMD. De Laat et al. demonstrated that, in patients with TMD, sore regions, especially in the m. sternocleidomastideus, are more common in comparison to patients without TMD [32]. M. masseter, temporalis, and pterygoid medialis can be painful and may also be swollen or hypertrophied. In certain patients, visible changes may be apparent without the need for imaging. However, if precise measurements of muscle thickness are required, such as before and after treatment, US [33] and MRI [34] are valuable imaging modalities. US is sufficient to measure the muscle thickness in certain parts, while MRI is offered to assess the complete muscle volume.

Often, the insertion or origin of the muscle is painful. Thus, it is worth considering the involvement of the deep part of the muscle m. masseter. In the common anatomy books, the m. masseter is shown as a muscle with superficial and deep layers. Mezey et al. described a third layer, which is found “running from the medial surface of the zygomatic process of the temporal bone to the root and posterior margin of the coronoid process” [35]. This part is called “pars coronoideus” by the authors and seems to be the part of the masseter muscle that can retract the lower jaw [35]. Its normal functioning is important to ensure the stability of the TMJ and its involvement should also be considered in patients with clenching and bruxism.

Koob et al. postulated that a risk factor for pain in the masticatory muscle system is the clenching movement while holding the mouthpiece in place [15]. Hardening of the muscle or parts of the muscle can be felt, if very prominent, as knots in the muscle. Basically, pain in this muscle group can be treated similarly to other muscle pains. One treatment approach for these cases is the intake of supplements. An intake of 350 mg magnesium a day showed a significant reduction in muscle soreness in comparison to a placebo in young adults [36]. Muscle cramps and twitching are signs of calcium deficiency [37]; therefore, a sufficient calcium intake is of importance for divers. Although cramps in the legs are better known, cramps can also occur in the masticatory muscle system. Cramps or myospasms occur suddenly. A continuous contraction of the muscle with muscle shortening, leading to trismus (a locked jaw), can occur. The mandible might not be able to move in its normal range. The sudden onset of the symptoms differentiates the cramp or spasm from other masticatory muscle pain disorders and myalgia, which present a dull, persisting pain with tenderness and aching muscles. Interestingly, mastication myalgia is seen more often in young-to-middle-aged adults [37].

Although NSAID treatment can help, the use of muscle relaxants should be considered very carefully. An individual’s ability to dive is not possible while taking muscle relaxants because of their sedative effects, with drowsiness as a side effect. Dizziness can also occur [38]. All of these conditions can be life-threatening, especially under water.

Other treatment options for TMDs of muscular origin vary enormously. The following items were assessed in the review and meta-analysis published by Al Moraissi et al.: counselling therapy, occlusal appliances, manual therapy, laser therapy, myofascial trigger point therapy and dry needling, intramuscular injection of local anaesthesia or botulinum toxin-A (BTX-A), muscle relaxants, hypnosis/relaxation therapy; oxidative ozone therapy, and placebo or no treatment [39]. The effective treatments are ranked as follows: manual therapy > counselling > local anaesthesia > occlusal appliances. However, the authors emphasized that the study situation is not very extensive, and conclusions need to be drawn cautiously [39].

Diving is reported to have a beneficial effect on the diver’s mood and on their mental health [40]. Patients with chronic masticatory muscle pain are more likely to have psychological symptoms [41]. Mental health issues, such as depression or anxiety, increase the probability of developing muscle tenderness [42]. In fact, an increase in masticatory muscle activity can be due to anxiety and stress, which can also lead to an overload of the TMJ [43]. Therefore, diving physicians should carefully examine divers with muscle pain and include assessments of their mental health.

A limitation of this study is evidently the small number of physicians we consulted to evaluate the algorithm. However, since it is not an experimental study and we obtained all relevant information, this limitation does not significantly detract from its validity. Expanding the algorithm would have increased its complexity for daily use; its advantage lies in its manageable size, making it easily printable and accessible. It is intended for use in daily practise or in settings where dentists or maxillofacial surgeons are not readily available. Nonetheless, for more severe pathologies, a referral cannot be avoided.

Another limitation pertains to the number of exercises included. We opted to showcase only a select few, focusing on their relevance. These exercises can complement other treatment approaches, especially for patients who are away from home e.g. on a dive boat. However, if the treatment options and exercises presented are deemed insufficient, a referral to a physiotherapist should be considered.

Future research endeavours in the realm of jaw pain among divers are underway. With the advent of new tools such as advanced three-dimensional (3D) US or US shear wave elastography (SWE) [44], conducting projects in this field will become more feasible and may uncover new insights. Another intriguing prospect is the utilization of artificial intelligence (AI), which is still in its nascent stages. Algorithms will gain more and more importance and could serve as a foundation for future research in this domain, offering promising avenues for exploration. 

## 5. Conclusions

The “jaw pain in a diver” can have various causes, but it can also be treated by numerous approaches. Our algorithm will be useful for diving physicians to make an accurate diagnosis. With this diagnosis, further treatment can be planned or a referral to a specialist (e.g., dentist, maxillofacial surgeon, orthodontics) can be initiated.

## Data Availability

The raw data supporting the conclusions of this article will be made available by the authors on request.

## Abbreviations

AI	Artificial intelligence
CBCT	Cone beam computed tomography
CT	Computed tomography
DCS	Decompression sickness
MRI	Magnetic resonance imaging
NSAIDs	Nonsteroidal anti-inflammatory drugs
SWE	Shear wave elastography
TMD	Temporomandibular disease
TMJ	Temporomandibular joint
3D	Three-dimensional
US	Ultrasound
